# Bullous pemphigoid and its association with neurological diseases

**DOI:** 10.4102/jcmsa.v2i1.27

**Published:** 2024-02-28

**Authors:** Charl P. Smit, Nicola A. Gray, Willem I. Visser

**Affiliations:** 1 Department of Dermatology, Faculty of Medicine and Health Sciences, Stellenbosch University, Stellenbosch, South Africa

**Keywords:** bullous pemphigoid, neurological disease, comorbidities, stroke, dermatology

## Abstract

**Background:**

Bullous Pemphigoid (BP) is the most common immunobullous skin disease.

**Methods:**

This was a descriptive cross-sectional study of electronic health records data at a specialist dermatology clinic at Tygerberg Hospital in the Western Cape. Patients presenting during a predefined 5-year period with a clinicopathologic diagnosis of BP were included.

**Results:**

The final sample comprised 54 cases of BP. The median age at diagnosis was 71.0 years, and 64.8% were female. Eighty per cent had at least one chronic medical condition at the time of BP diagnosis. The most common comorbidities were hypertension (63.0%), diabetes mellitus (33.3%) and strokes (24.1%). Twenty-eight per cent had been diagnosed with at least one neurological comorbidity at the time of BP diagnosis. Patients experienced a median delay of 45 days (interquartile range: 21.0-90.0 days) between onset of symptoms and presentation to dermatology.

**Conclusion:**

This is the first study reporting co-morbidities in patients with BP in sub-Saharan Africa. Despite the high burden of disease due to physical injury in the study setting, no patients with BP had a history of traumatic brain injury. This suggests the possibility that the association between BP and neurologic disorders is limited to stroke, epilepsy, and neurodegenerative diseases. Future prospective cohort studies are indicated to further investigate the results of this study.

**Contribution:**

To describe neurologic and other comorbidities in patients presenting with BP at a tertiary hospital in South Africa. This research in South Africa, a lower-middle-income country, seeks to address knowledge gaps, utilize data, and advance the field of dermatology.

## Introduction

Bullous pemphigoid (BP) is the most common autoimmune bullous skin disease. It mainly affects individuals over the age of 65 years, and typically presents with tense bullae on an erythematous, urticarial base.^1^ Clinically observed blistering correlates histologically to sub-epidermal cleavage, caused by autoantibodies directed against hemidesmosomal proteins BPAG2 (BP180 or type XVII collagen) and BPAG1 (BP230) at the dermo-epidermal junction.^2^

The annual incidence of BP is approximately 2.4–21.7 per million across most regions of the world, including France, Switzerland, Germany, among others.^1^ An increasing incidence of BP has been reported in various European countries. Potential explanations include increased life expectancy and improved clinical recognition of BP variants, among others.^1^ Bullous pemphigoid is associated with impaired quality of life as measured by the Dermatology Life Quality Index (DLQI) and an increased risk of depression and loneliness.^3^ Furthermore, BP is associated with an increased risk of mortality, with Standardized Mortality Ratios (SMRs) ranging from 1.9–7.2 across studies.^1^

Systematic review evidence suggests a statistically significant association between BP and neurologic diseases, specifically stroke, Parkinson’s disease, dementia, epilepsy, and multiple sclerosis.^2^ The data on which this systematic review is based were predominantly from Taiwan, Europe, and the United States (US), and no African countries were represented.

The relationship between BP and other comorbidities has also been studied. Meta-analysis shows no association between BP and ischaemic heart disease.^4^ The relationship between BP and malignancy remains uncertain. Meta-analyses show a statistically significant relationship between BP and haematologic malignancies, but not between BP and solid malignancies.^5^ Meta-analysis also indicates that the use of certain medications is associated with increased odds of BP, specifically aldosterone antagonists, dipeptidyl peptidase 4 inhibitors, anticholinergics, and dopaminergic medications.^6^ Once again, the data on which these systematic reviews are based does not include African countries.

South Africa is an upper-middle-income country,^7^ and many complex social and economic factors contribute to a unique burden of disease in the country. The burden of disease includes communicable diseases (e.g., human immunodeficiency virus [HIV] and tuberculosis [TB]), non-communicable diseases (e.g., stroke and ischaemic heart disease), poverty-related conditions (e.g., diarrhoeal diseases and low birth weight), and physical injuries (e.g., homicide and road traffic accidents).^8,9^ It is therefore possible that pre-existing co-morbidities in patients with BP in South Africa differ from other settings.

The aim of this study was to describe the comorbidities in patients presenting to a tertiary level dermatology clinic with BP in Cape Town, South Africa. To the best of our knowledge, this is the first such study from sub-Saharan Africa.

## Research methods and design

This descriptive cross-sectional study involved a retrospective review of medical records at Tygerberg Hospital (Cape Town, South Africa). Patients were included if they presented between 01 January 2016 until 31 December 2020 with a final diagnosis of BP made, based on both clinical and histopathologic findings. There were no age or sex restrictions. Patients were excluded if the diagnosis was not supported by histopathologic findings, including direct immunofluorescence.

The following variables are reported: demographic characteristics (age and sex), time between onset of symptoms and diagnosis of BP, and pre-existing comorbidities at the time of BP diagnosis. For continuous variables with normal distributions, averages are presented as means with standard deviations (s.d). For skewed distributions, averages are presented as medians with interquartile ranges (IQRs).

### Ethical considerations

The Health Research Ethics Committee (HREC) of Stellenbosch University granted ethical clearance for this study (Project ID: 24771; Ethics Reference Number: U22/03/160). As this is a retrospective study, a waiver of informed consent was deemed appropriate. Confidentiality was maintained through data that was anonymised at the point of data capture.

## Results

A total of 57 potentially eligible patients were identified from medical records. Three cases were excluded because the diagnosis of BP in each was subsequently revised to bullous systemic lupus erythematosus, bullous scabies and epidermolysis bullosa acquisita, respectively. The demographic characteristics of the 54 remaining patients included in the final analysis are shown in Table 1. The median age at the time of BP diagnosis was 71.0 years (IQR: 65.3–82.0 years). No patients diagnosed with BP during this 5-year period were under the age of 20 years. Of the total patients (*n* = 54), 19 (35.2%) were male and 35 (64.8%) were female. In the subgroup of patients over the age of 75 years, 19 out of 24 (79%) were female. The median duration of symptoms prior to diagnosis of BP was 45.0 days (IQR: 21.0–90.0 days).

**TABLE 1:** Baseline characteristics of patients with bullous pemphigoid.

Characteristic	Value			
	Median	IRQ	*n*	%
Age in years at diagnosis	71.0	65.3–82.0	-	-
**Age group at diagnosis (years)**				
< 20	-	-	0	0.0
20–49	-	-	2	3.7
50–59	-	-	7	13.0
60–69	-	-	13	24.1
70–79	-	-	12	22.2
≥ 80	-	-	20	37.0
**Sex**				
Male	-	-	19	35.2
Female	-	-	35	64.8
Duration of symptoms prior to diagnosis	45	21–90	-	-

IQR, interquartile range.

### Comorbidities associated with bullous pemphigoid

Forty-three patients (79.6%) had at least one chronic disease at the time of BP diagnosis. The most prevalent pre-existing systemic diseases were hypertension (*n* = 34, 63.0%), type 2 diabetes mellitus (*n* = 18, 33.3%) and strokes (*n* = 13, 24.1%) (Table 2). Fifteen patients (27.8%) had at least one type of pre-existing neurological disease. Stroke was the most common neurological disease (*n* = 13, 24.1%), followed by dementia (*n* = 1, 2%) and epilepsy (*n* = 1, 2%). Out of all the BP patients, only 3 patients (6%) had a known malignancy. These were bronchial, prostate and breast carcinoma. None of the patients with BP had a history of traumatic brain injury (TBI).

**TABLE 2:** Pre-existing comorbidities at time of bullous pemphigoid diagnosis (*N* = 54).

Disease	Patients with BP	
	*n*	%
Neurological disease	15	27.8
Stroke	13	24.1
Dementia	1	1.9
Epilepsy	1	1.9
Parkinsons	0	0.0
Multiple sclerosis	0	0.0
Cardiovascular	41	76.8
Hypertension	34	63.0
Dyslipidemia	7	13.0
Ischaemic heart disease	0	0.0
Arrhythmia	0	0.0
Endocrinopathies	20	37.0
Diabetes mellitus	18	33.3
Hypothyroidism	2	3.7
Psychiatric diagnoses	4	7.4
Depression	2	3.7
Other mood disorders	2	3.7
Other		
COPD	4	7.4
Overweight or obese (BMI > 25)	2	3.7
Malignancies	3	5.6
Renal disease	2	3.7
HIV	3	5.6

BP, bullous pemphigoid; COPD, chronic obstructive pulmonary disease; BMI, body mass index; HIV, human immunodeficiency virus.

## Discussion

This descriptive, cross-sectional study reports comorbidities in patients presenting to a tertiary hospital dermatology clinic with BP over a 5-year period. Firstly, in terms of baseline characteristics, we found that most of our patients were female (64.8%) and the median age at presentation was 71.0 years. Secondly, we found that 81.5% of patients had at least one chronic medical condition at the time of BP diagnosis. The most common preceding chronic medical conditions were hypertension (63.0%), type 2 diabetes mellitus (33.3%) and stroke (24.1%). Thirdly, we found that 27.8% of our sample had a pre-existing neurological disease, of which stroke was overwhelmingly the most common. No patients had a history of TBI. Lastly, although not a primary objective of the study, it was notable that there was a median delay of 45 days (IQR: 21.0–90.0 days) between onset of symptoms and presentation to dermatology.

The female predominance and advanced age of patients with BP in our study are in keeping with observations in other settings. In our study 35 of 54 participants (64.8%) were female, equating to a female-to-male ratio of 1.8. Similarly, in other studies, female-to-male ratios range from 1.04–5.1.^1^ It has been suggested that the incidence of BP in women is only higher than in men up until the age of 75 years, after which time the incidence is higher in men.^1^ Our study did not support this, as in the subgroup of patients over the age of 75 years, 19 out of 24 (79%) were female. The median age of our participants was 71.0, with half of our cohort falling between the ages of 65.3 and 82.0 years. This is consistent with other settings, where the mean age of presentation with BP is reported to be between 66.0 and 83.0 years.^1^

Sixty-three per cent of BP patients in this study had a preceding diagnosis of hypertension. This is higher than the proportion of BP patients with hypertension reported in other settings, for example Finland (44.0%), Korea (32.1%) and Turkey (43.4%).^10,11,12^ However, the higher rates of hypertension in BP patients in this study could simply reflect the high prevalence of hypertension in older adults in South Africa. For example, one national population-based cross-sectional study in South Africa reported hypertension in 77.3% of adults 50 years and older (*n* = 3840).^13^

A third of BP patients in this study had a preceding diagnosis of type 2 diabetes mellitus. As this study did not have a control group, it is not possible to ascertain whether there is a correlation between BP and type 2 diabetes. However, it is possible that the high prevalence of type 2 diabetes in this study simply mirrors the high prevalence of diabetes in older adults in South Africa. According to the results of a systematic review and meta-analysis, the prevalence of type 2 diabetes in South Africa across all age groups is 15.25% (95% confidence interval [CI]: 11.07–19.95).^14^ Sub-group analysis showed that the prevalence increases with age, and with three included studies reporting a prevalence above 30% in those over the age of 65 years. This is similar to the proportion of BP patients with diabetes mellitus reported in other settings for example Finland (34.0%), Korea (34.8%) and Turkey (31.7%).^10,11,12^ Gliptins, also known as dipeptidyl peptidase-4 inhibitors (DPP-4i), are associated with the development of BP.^6,15^ However, this class of glucose-lowering drug is not yet routinely available in our setting and none of the patients in this study had prior exposure to Gliptins.

In all 15 patients with BP (27.8%) had a previously diagnosed neurological disease. Most of these (13 of 15) were strokes. The prevalence of stroke in our study is higher than the purported prevalence in South Africa of 1.29% (95% CI: 0.83–1.75).^16^ Although the national prevalence of stroke according to age is not known, it seems probable that the prevalence of stroke in BP patients in this study is higher than that of the average age-adjusted population. Systematic review evidence indicates that patients with BP have an increased risk of neurologic disorders (risk ratio [RR]: 4.93, 95% CI: 3.62–6.70, I^2^ = 50.6%).^2^ BPAG1 and BPAG2 are expressed in both the skin and brain. It is hypothesised that neurological diseases may expose the immune system to neural isoforms of BP antigens, leading to loss of immune privilege. The resultant autoantibodies then cross-react with BP antigens in the skin, resulting in the development of BP.^2^

The proportion of BP patients with neurological disease in our study (27.8%) is lower than reported in several other studies for example from Finland (46%), France (36%), and Portugal (55.8%).^10,17,18^ There are, however, several studies reporting lower proportions of neurological disease, for example from Turkey (19.3%), Iran (19.5%), and the US (23.0%).^12,19,20^ There are two facts that raise the possibility that neurologic disorders are under-diagnosed and/or under-reported in the present study. Firstly, despite the high prevalence of hypertension and diabetes mellitus discussed above, no patients were diagnosed with transient ischaemic attacks (TIA). It is probable that patients did not volunteer this medical history because of the resolution of symptoms. Secondly, there was only one patient with dementia, despite this being in the top 2 most frequently reported neurologic comorbidities in the abovementioned studies.^10,12,17,18,19^ Screening for cognitive impairment is not done routinely in the outpatient dermatology clinic, and patients with more subtle diseases might have remained undiagnosed.

South Africa has an exceptionally high burden of physical injuries, including interpersonal violence and road traffic accidents.^21^ Given this setting, it is notable that none of the patients with BP in the present study had a history of TBI. It therefore seems unlikely that TBI is a risk factor for BP. This suggests the possibility that the association between BP and neurologic disorders is limited to stroke, epilepsy, and neurodegenerative diseases.

Although not one of our primary objectives, we observed that patients experienced considerable delay in diagnosis. The median waiting time was 45 days, with half of the cohort waiting between 21 days and 90 days. Given the increasing prevalence and significant morbidity and mortality associated with BP, the reasons for this delay should be investigated and addressed to improve the quality of care.

### Limitations

This study had several limitations. Firstly, as this was a retrospective study that relied on medical records, some comorbidities may have been missed. Detailed records of chronic medications used at the time of onset of BP were also not consistently available. Secondly, as this was an uncontrolled study, relative risks could not be calculated. Thirdly, records only extended to patients seen in the dermatology outpatient’s clinic. Patients who were diagnosed via inpatient referrals who did not attend follow-up appointments might have been missed. Fourthly, our sample consisted of only 54 patients from a single province in South Africa. Although, as discussed above, BP is a rare disease with an incidence of approximately 2.4–21.7 per million, selection bias remains a concern. Without more data, limited inferences can be made about comorbidities in sub-Saharan Africans with BP in general.

## Conclusion

Results from this study found that most patients (80%) presenting to a tertiary hospital dermatology clinic with BP over a 5-year period had at least one comorbidity. The most common comorbidities are hypertension (63%), type 2 diabetes mellitus (33%), and stroke (24%). Despite the high burden of injuries in South Africa, no patients with BP had a history of TBI. Patients experienced considerable delay in diagnosis, with a median wait of 45 days (IQR: 21.0–90.0 days) between the onset of symptoms and presentation to dermatology. This is the first study reporting on comorbidities in patients with BP in sub-Saharan Africa. Future prospective cohort studies are indicated to further investigate these associations, as well as to evaluate potentially implicated drugs. Data from other referral centres in sub-Saharan Africa would improve the generalisability of findings. The reasons for diagnostic delay also warrant further study.

## References

[1] Kridin K. Subepidermal autoimmune bullous diseases: Overview, epidemiology, and associations. Immunol Res. 2018;66(1):6–17. 10.1007/s12026-017-8975-2 29159697

[2] Lai YC, Yew YW, Lambert WC. Bullous pemphigoid and its association with neurological diseases: A systematic review and meta-analysis. J. Eur Acad Dermatol Venereol. 2016;30(12):2007–2015. 10.1111/jdv.13660 27599898

[3] Kouris A, Platsidaki E, Christodoulou C. et al. Quality of life, depression, anxiety and loneliness in patients with bullous pemphigoid. A case control study. An Bras Dermatol. 2016;91(5):601–614. 10.1590/abd1806-4841.20164935 27828632 PMC5087217

[4] Lai CY, Lin MH, Tsai HH, Chang HC. Association between bullous pemphigoid and ischemic heart diseases: A systematic review and meta-analysis. J Am Acad Dermatol. 2020;83(3):938–940. 10.1016/j.jaad.2020.01.032 31982402

[5] Atzmony L, Mimouni I, Reiter O. et al. Association of bullous pemphigoid with malignancy: A systematic review and meta-analysis. J Am Acad Dermatol. 2017;77(4):691–699. 10.1016/j.jaad.2017.05.006 28645646

[6] Liu SD, Chen WT, Chi CC. Association between medication use and bullous pemphigoid: A systematic review and meta-analysis. JAMA Dermatol. 2020;156(8):891–900. 10.1001/jamadermatol.2020.1587 32584924 PMC7301306

[7] Joseph C. Developing an Inclusive South Africa. Washington, D.C.: World Bank; 2018.

[8] Bradshaw D, Groenewald P, Laubscher R. et al. Initial burden of disease estimates for South Africa, 2000. S Afr Med J. 2003;93(9):682–688. 14635557

[9] Achoki T, Sartorius B, Watkins D, et al. Health trends, inequalities and opportunities in South Africa’s provinces, 1990–2019: Findings from the Global Burden of Disease 2019 Study. J Epidemiol Community Health. 2022;76(5):471–481. 10.1136/jech-2021-217480 35046100 PMC8995905

[10] Pankakoski A, Sintonen H, Ranki A, Kluger N. Comorbidities of bullous pemphigoid in a Finnish cohort. Eur J Dermatol. 2018;28(2):157–161. 10.1684/ejd.2018.3243 29619987

[11] Hye Lee J, Kim S-C. Mortality of patients with bullous pemphigoid in Korea. J Am Acad Dermatol. 2014;71(4):676–683. 10.1016/j.jaad.2014.05.006 24930586

[12] Kılıç Sayar S, Sun GP, Küçükoğlu R. Comorbidities of bullous pemphigoid: A single-center retrospective case-control study from Turkey. Dermatol Ther. 2021;34(5):e15031. 10.1111/dth.15031 34137146

[13] Peltzer K, Phaswana-Mafuya N. Hypertension and associated factors in older adults in South Africa. Cardiovasc J Afr. 2013;24(3), 67–72. 10.5830/CVJA-2013-002 23736129 PMC3721893

[14] Pheiffer C, Wyk VPV, Turawa E, Levitt N, Kengne AP, Bradshaw D. Prevalence of type 2 diabetes in South Africa: A systematic review and meta-analysis. Int J Environ Res Public Health. 2021;18(11):5868. 10.3390/ijerph18115868 34070714 PMC8199430

[15] Chouchane K, Di Zenzo G, Pitocco D, Calabrese L, De Simone C. Bullous pemphigoid in diabetic patients treated by gliptins: The other side of the coin. J Transl Med. 2021;19:520. 10.1186/s12967-021-03192-8 34930319 PMC8691092

[16] Abdelatif N, Peer N, Manda SO. National prevalence of coronary heart disease and stroke in South Africa from 1990–2017: A systematic review and meta-analysis. Cardiovasc J Afr. 2021;32(3):156–160. 10.5830/CVJA-2020-045 33769427 PMC8756070

[17] Cordel N, Chosidow O, Hellot M.-F. et al. Neurological disorders in patients with bullous pemphigoid. Kargar. 2007;215(3), 187–191. 10.1159/000106574 17823513

[18] Teixeira VB, Cabral R, Brites MM, Vieira R, Figueiredo A. Bullous pemphigoid and comorbidities: A case-control study in Portuguese patients. An Bras Dermatol. 2014;89(2):274–278. 10.1590/abd1806-4841.20142516 24770504 PMC4008058

[19] Khosravani S, Handjani F, Alimohammadi R, Saki N. Frequency of neurological disorders in bullous pemphigoid patients: A cross-sectional study. Int Sch Res Notices. 2017;2017:6053267 10.1155/2017/6053267 28630891 PMC5463116

[20] Brick KE, Weaver CH, Savica R, et al. A population-based study of the association between bullous pemphigoid and neurologic disorders. J Am Acad Dermatol. 2014;71(6):1191–1197. 10.1016/j.jaad.2014.07.052 25174542 PMC4326088

[21] Norman R, Matzopoulos R, Groenewald P, Bradshaw D. The high burden of injuries in South Africa. Bull World Health Organ. 2007;85(9):695–702. 10.2471/blt.06.037184 18026626 PMC2636399

